# Comparison of four COVID-19 screening strategies to facilitate early case identification within the homeless shelter population: A structured summary of a study protocol for a randomised controlled trial

**DOI:** 10.1186/s13063-020-04890-2

**Published:** 2020-11-23

**Authors:** Timothy O’Shea, Lawrence Mbuagbaw, Vaibhav Mokashi, David Bulir, Jodi Gilchrist, Nicole Smieja, Sylvia Chong, Sarah Marttala, Valentina Vera, Anna Cvetkovic, Marek Smieja

**Affiliations:** 1 Department of Medicine (Infectious Diseases), Hamilton Shelter Health Network, Juravinski Hospital & Cancer Centre, McMaster University, A3-66, 711 Concession Street, Hamilton, ON L8V 1C3 Canada; 2 Department of Health Research, Faculty of Health Sciences, Methods, Evidence and Impact, McMaster University, Hamilton, Canada; 3 Department of Medicine (Infectious Diseases), McMaster University, Hamilton, Canada; 4 Department of Medicine, Pathology and Molecular Medicine, Research St. Joseph’s – Hamilton, McMaster University, Hamilton, ON Canada; 5 Research St. Joseph’s – Hamilton, Hamilton, ON Canada

**Keywords:** COVID-19, Randomized trial, protocol, screening, homeless, shelter, swabs, prevention

## Abstract

**Objectives:**

1. To compare the effectiveness of four different surveillance strategies in detecting COVID-19 within the homeless shelter population.

2. To assess the participant adherence over time for each surveillance method.

**Trial Design:**

This is a prospective cluster-randomized study to compare the effectiveness of four different surveillance regimens across eight homeless shelters in the city of Hamilton.

**Participants:**

Participants will include both residents of, and the staff working within, the homeless shelters. All participants aged 18 or older who consent to the study and are able to collect a swab sample (where relevant) are eligible for the study. The study will take place across eight homeless shelters (four men-only and four women-only) in the City of Hamilton in Ontario, Canada.

**Intervention and Comparator Groups:**

The comparator group will receive active daily surveillance of symptoms and testing will only be completed in symptomatic participants (i.e. those who fail screening or who seek care for potential COVID-19 related symptoms).

The three intervention arms will all receive active daily surveillance of symptoms and testing of symptomatic participants (as in the comparator group) in addition to one of the following:

1. Once weekly self-collected oral swabs (OS) regardless of symptoms using written and visual instructions.

2. Once weekly self-collected oral-nares swab (O-NS) regardless of symptoms using written and visual instructions.

3. Once weekly nurse collected nasopharyngeal swab (NPS) regardless of symptoms.

Participants will follow verbal and written instructions for the collection of OS and O-NS specimens. For OS collection, participants are instructed to first moisten the swab on their tongue, insert the swab between the cheek and the lower gums and rotate the swab three times. This is repeated on the other side. For O-NS collection, after oral collection, the swab is inserted comfortably (about 2-3 cm) into one nostril, parallel to the floor and turned three times, then repeated in the other nostril. NPS specimens were collected by the nurse following standard of care procedure.

All swabs were placed into a viral inactivation medium and transported to the laboratory for COVID-19 testing. Briefly, total nucleic acid was extracted from specimens and then amplified by RT-PCR for the UTR and Envelope genes of SARS-CoV-2 and the human RNase P gene, which is used as a sample adequacy marker.

**Main Outcomes:**

1. Primary outcome: COVID-19 detection rate, i.e. the number of new positive cases over the study period of 8 weeks in each arm of the study.

2. Secondary outcomes: Qualitative assessment of study enrollment over 8 weeks. Percentage of participants who performed 50% or more of the weekly swabs in the intervention arms in the 8 week study period.

**Randomization:**

We will use a computer-generated random assignment list to randomize the shelters to one of four interventions. Shelters were stratified by gender, and the simple randomization scheme was applied within each stratum. The randomization scheme was created using WinPEPI.

**Blinding:**

This is an open-label study in which neither participants nor assessors are blinded.

**Numbers to be randomized (sample size):**

Since we are including our total sample frame, a sample size estimation at the cluster level is not required. However, if we succeed to enroll 50 participants per shelter from 8 shelters (*n*=400), and the detection rate is 3 times higher in the intervention groups (0.15) than in the comparator groups (0.05), we will have 90% power to detect a statistically significant and clinically important difference at a type I error rate of alpha=0.05 (one tailed), assuming an intraclass correlation of ~0.008. These computations were done using WinPEPI, and informed by conservative estimates from other studies on respiratory illness in the homeless (see Full protocol).

**Trial Status:**

The protocol version number is 3.0. Recruitment began on April 17, 2020 and is ongoing. Due to low numbers of COVID cases in the community and shelter system during the initial study period, the trial was extended. The estimated date for the end of the extended recruitment period is Feb 1, 2021.

**Trial Registration:**

The trial was registered with ClinicalTrials.gov on June 18, 2020 with the identifier NCT04438070.

**Full protocol:**

The full protocol is attached as an additional file, accessible from the Trials website (Additional file 1). In the interest in expediting dissemination of this material, the familiar formatting has been eliminated; this Letter serves as a summary of the key elements of the full protocol.

**Supplementary Information:**

**Supplementary information** accompanies this paper at 10.1186/s13063-020-04890-2.

## Supplementary Information

**Additional file 1.** Full study protocol

## Data Availability

Not applicable

