# Supplementary material for: Comparison of four COVID-19 screening strategies to facilitate early case identification within the homeless shelter population: A structured summary of a study protocol for a randomised controlled trial
Source: Trials. 2020 Nov 23;21:941. doi: 10.1186/s13063-020-04890-2 (PMC7680991; doi:10.1186/s13063-020-04890-2)
Supplement: Supplementary file 1 — Additional file 1. Full study protocol [file 13063_2020_4890_MOESM1_ESM.docx]

**Comparison of four COVID-19 screening strategies to facilitate early case identification within the homeless shelter population**

## **Authors**

Timothy O’Shea MD, MPH (Corresponding Author)

Department of Medicine (Infectious Diseases), McMaster University

Hamilton Shelter Health Network

Juravinski Hospital & Cancer Centre

A3-66, 711 Concession Street

Hamilton, ON, Canada L8V 1C3

Phone: (905) 389-4411 Ext. 42471

[osheat@mcmaster.ca](mailto:osheat@mcmaster.ca)

Lawrence Mbuagbaw MD, MPH, PhD
Faculty of Health Sciences, Department of Health Research, Methods, Evidence and Impact, McMaster University
[mbuagblc@mcmaster.ca](mailto:mbuagblc@mcmaster.ca)

Vaibhav Mokashi BSc, MD

Department of Medicine (Infectious Diseases), McMaster University

[Vaibhav.mokashi@medportal.ca](mailto:Vaibhav.mokashi@medportal.ca)

David Bulir MD, PhD

Department of Medicine, Pathology and Molecular Medicine, McMaster University

Research St. Joseph’s – Hamilton

Hamilton, ON

[bulirdc@mcmaster.ca](mailto:bulirdc@mcmaster.ca)

Jodi Gilchrist MSc, CCRP
Research St. Joseph’s – Hamilton
Hamilton, ON

[jgilchri@stjosham.on.ca](mailto:jgilchri@stjosham.on.ca)

Nicole Smieja BA

Research St. Joseph’s – Hamilton

Hamilton, ON

[nsmieja@stjosham.on.ca](mailto:nsmieja@stjosham.on.ca)

Sylvia Chong BSc

Research St. Joseph’s – Hamilton

Hamilton, ON

[schong@stjosham.on.ca](mailto:schong@stjosham.on.ca)

Sarah Marttala

Research St. Joseph’s – Hamilton

Hamilton, ON

[smarttal@stjosham.on.ca](mailto:smarttal@stjosham.on.ca)

Valentina Vera

Research St. Joseph’s – Hamilton

Hamilton, ON

vvera@stjosham.on.ca

Anna Cvetkovic

Department of Medicine (Infectious Diseases), McMaster University

[anna.cvetkovic@medportal.ca](mailto:anna.cvetkovic@medportal.ca)

Marek Smieja MD, PhD

Department of Medicine, Pathology and Molecular Medicine, McMaster University

Hamilton, Canada
[smiejam@mcmaster.ca](mailto:smiejam@mcmaster.ca)

## **Background**

There are currently no observational or randomized studies aimed at determining the optimal strategy for surveillance and outbreak prevention in the homeless shelter setting despite a history of well documented outbreaks with various pathogens including tuberculosis and respiratory viruses (1,2). Determining an optimal strategy is important in order to prevent outbreaks in high risk settings especially in the context of a viral pandemic such as the current SARS-CoV-2 pandemic. Outbreak prevention will decrease the mortality and morbidity associated with viral respiratory. This will naturally be associated with decreased healthcare related costs. Furthermore, surveillance for respiratory viruses, especially in the context of a pandemic, may empower a generally marginalized population.

## **Study Design**

We are conducting a prospective random cluster-allocation study to compare the effectiveness of four different surveillance regimens across eight shelters in Hamilton (four men’s and four women’s shelters). The shelters are associated with Good Shepherd, Mission Services, Wesley Urban Ministries, YWCA and Hamilton Native Women’s Centre; all have agreed to work with us on this study. The surveillance methods are as follows:

1. Active daily surveillance of symptoms and testing only symptomatic participants. This is the current standard of care protocol for residents accessing the shelters.
2. Once weekly self-collected oral swabs (OS) regardless of symptoms using written and visual instructions (attached).
3. Once weekly self-collected oral-nares swab (O-NS) regardless of symptoms using written instructions (attached).
4. Once weekly nurse collected nasopharyngeal swab (NPS) regardless of symptoms


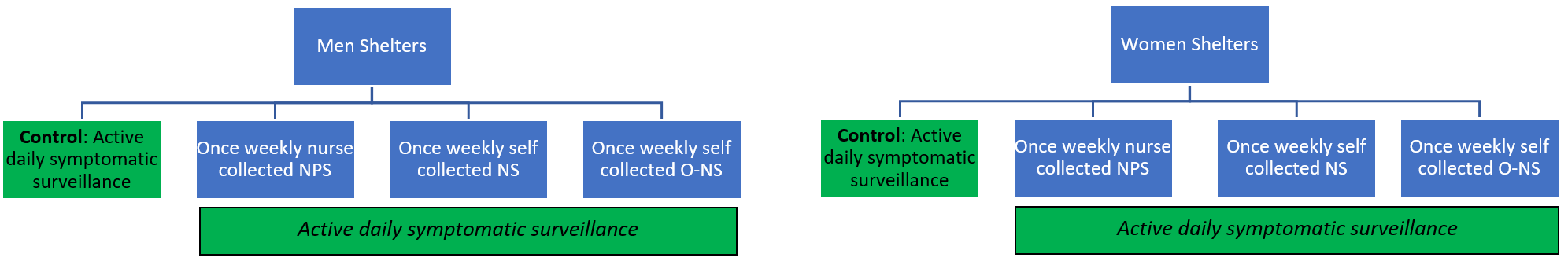

All participants in the intervention groups will continue to receive active daily surveillance of symptoms. Only participants who provide consent will be considered in the intervention groups and verbal consent will be recorded by study staff at each site. At all sites participants who seek care for respiratory symptoms or symptoms potentially related to COVID-19 would get testing as is routinely done.

Each surveillance regimen will occur for 6-8 weeks based on the funding available. Each shelter has approximately 100 participants and we are aiming to enroll 30-50% of participants at each site.

## **Outcomes**

1. Primary outcome: COVID-19 detection rate, i.e. the number of new positive cases over the study period in each arm of the study.
2. Secondary outcomes: Qualitative assessment of study enrollment over time. Percentage of participants who performed 50% or more of the weekly swabs in the intervention arms.

## **Sample size considerations**

Since we are including our total sample frame, a sample size estimation at the cluster level is not required. However, if we succeed to enroll 50 participants per shelter from 8 shelters (n=400), and the detection rate is 3 times higher in the intervention groups (0.15) than in the comparator groups (0.05), we will have 90% power to detect a statistically significant and clinically important difference at a type I error rate of alpha=0.05 (one tailed), assuming an intraclass correlation of ~0.008. These computations were done using WinPEPI, and informed by conservative estimates from other studies on respiratory illness in the homeless (4, 5).

## **Inclusion Criteria:**

1. Willing and able to provide verbal consent to participation in the study.
2. Male or female > 18 years of age, residing in one of the homeless shelters within Hamilton, ON.

## **Exclusion Criteria:**

1. Unable or unwilling to give consent.
2. Male or female < 18 years of age.

## **Data Collection Methods**

1. COVID-19 swab results will be processed as per usual protocol by the Virology Research Lab at St. Joseph’s Healthcare (SJH)

Specimens identified as positive in the research lab will be directed to the Clinical Virology Lab within HRLMP for confirmatory testing.

1. All swabs were placed into a viral inactivation medium and transported to the laboratory for COVID-19 testing. Briefly, total nucleic acid was extracted from specimens and then amplified by RT-PCR for the UTR and Envelope genes of SARS-CoV-2 and the human RNase P gene, which is used as a sample adequacy marker.

## **References**

1. McElroy PD, Southwick KL, Fortenberry ER, Levine EC, Diem LA, Woodley CL, et al. Outbreak of Tuberculosis Among Homeless Persons Coinfected with Human Immunodeficiency Virus. Clin Infect Dis. 2003 May 15;36(10):1305–12.
2. Tjon GMS, Götz H, Koek AG, de Zwart O, Mertens PLJM, Coutinho RA, et al. An outbreak of hepatitis A among homeless drug users in Rotterdam, The Netherlands. J Med Virol. 2005 Nov;77(3):360–6.
3. Betsch C, Wieler LH, Habersaat K. Monitoring behavioural insights related to COVID-19. The Lancet. 2020 Apr.

Wrezel O. Respiratory infections in the homeless. *UWO Med J* 2009; **78**(2): 61-5.

1. Thiberville S-d, Salez N, Benkouiten S, Badiaga S, Charrel R, Brouqui P. Respiratory viruses within homeless shelters in Marseille, France. *BMC research notes* 2014; **7**(1): 81.

INSTRUCTIONS FOR ORAL SELF-COLLECTION

*Due to copyright issues, the pictures have not been published.*

| **1)** | Grasp red handle, remove swab from plastic tube, and place tube on a clean surface. |  |
| --- | --- | --- |
| **2)** | Moisten swab by touching it to your tongue for a few seconds. |  |
| **3)** | Insert swab between cheek and lower gums. Roll the swab three times (3X). You may need to place your fingers on the outside of your cheek to help with sample collection. |  |
| **4)** | Repeat on the other cheek, rolling the swab three times (3X). |  |
| **5)** | Place swab back into plastic tube and close tightly. |  |

INSTRUCTIONS FOR ORAL-NASAL SELF-COLLECTION

*Due to copyright issues, the pictures have not been published.*

| **1)** | Grasp red handle, remove swab from plastic tube, and place tube on a clean surface. |  |
| --- | --- | --- |
| **2)** | Moisten swab by touching it to your tongue for a few seconds. |  |
| **3)** | Insert swab between cheek and lower gums. Roll the swab three times (3X). You may need to place your fingers on the outside of your cheek to help with sample collection. |  |
| **4)** | Repeat on the other cheek, rolling the swab three times (3X). |  |
| **5)** | Insert the swab comfortably into your nostril. Roll the swab around 3X, repeat on the other side. |  |
| **6)** | Place swab back into plastic tube and close tightly. |  |
